# The American and Colonial Journals: Medical Statistics

**Published:** 1839-01

**Authors:** 


### MEDICAL STATISTICS.

*Mortality Table for the City of New York, (with the Names of the Diseases,) for a Period of thirty-two years; viz. from* 1805 *to* 1836, *both inclusive.* By H. G. Dunnel, M.D., City Inspector.

| Diseases. | Total Deaths. | Comparative Mortality from each Disease.* |
|---|---|---|
| *Nervous System.* | | |
| Inflammation of Brain . | 1676 | 1 in 72. |
| Dropsy of Brain . . | 4986 | 1 in 24·2. |
| Apoplexy . . | 2075 | 1 in 58·11. |
| Palsy . . . | 1057 | 1 in 114·1. |
| Convulsions . . | 9343 | 1 in 12·91. |
| Epilepsy . . . | 219 | 1 in 551·07. |
| Catalepsy . . | 1 | |
| Hysteria . . . | 30 | 1 in 4022·8. |
| Chorea, or St. Vitus's Dance | 7 | 1 in 17240. |
| Asphyxia . . . | 74 | 1 in 1630·86. |
| Neuralgic, or Nervous Diseases | 100 | 1 in 1206·84. |
| Lock-jaw . . . | 191 | 1 in 631·85. |
| Hydrophobia . . | 11 | 1 in 10971. |
| Insanity . . . | 367 | 1 in 328·83. |
| *Respiratory System.* | | |
| Croup . . . . | 3947 | 1 in 30·57. |
| Hooping-cough . . | 2516 | 1 in 47·96. |
| †Lungs or Membr. Inflammation | 7578 | 1 in 15·92. |
| Dropsy of the Chest . | 883 | 1 in 136·67. |
| Bleeding from the Lungs . | 196 | 1 in 615·73. |
| Asthma . . . . | 328 | 1 in 367·93. |
| Consumption . . | 24883 | 1 in 4·85. |
| *Circulatory System.* | | |
| Heart, Organic Disease of . | 79 | 1 in 1527·64. |
| Fever, (type not named) . | 1475 | 1 in 81·81. |
| Intermittent . | 327 | 1 in 369. |
| Remittent and Bilious | 1480 | 1 in 81·51. |
| Malignant or Yellow | 477 | 1 in 253. |
| Inflammatory . | 143 | 1 in 843·94. |
| Typhus and Nervous | 3602 | 1 in 33·5. |
| Hectic . . | 59 | 1 in 2043. |
| *Eruptive Fevers.* | | |
| Scarlet . . . | 1983 | 1 in 60·85. |
| Measles . . . . | 2117 | 1 in 57. |
| Small-pox . . . | 2578 | 1 in 43·75. |

* No notice is taken in this column of Casualties, Suicides, and Still-births.

† Among the deaths from inflammation of the lungs and membranes are classed deaths reported by pleurisy, peripneumonia, bronchitis, cold, cough, influenza, pneumonia typhoides, and inflammation of the chest.

| Diseases. | Total Deaths. | Comparative Mortality from each Disease. |
|---|---|---|
| Water[?] . . . | 7 | 1 in 17240. |
| Erysipelas . . . | 289 | 1 in 417·23. |
| Herpes . . . | 20 | 1 in 6034·2. |
| Aphthæ, or Sprue . . | 802 | 1 in 150·47. |
| Leprosy . . . | 6 | 1 in 20114. |
| Scalded Head . . | 3 | 1 in 40228. |
| Aneurism . . | 56 | 1 in 2155·07. |
| Bleeding from parts not named | 328 | 1 in 367·81. |
| Dropsy of organs . | 3431 | 1 in 35·17. |
| *Digestive System.* | | |
| Stomach, bleeding from . | 21 | 1 in 5746·9. |
| Dyspepsia, or Indigestion | 122 | 1 in 907·24. |
| and Bowels, Inflammation of | 3252 | 1 in 37·11. |
| Cramp of . . | 241 | 1 in 500·76. |
| Colic . . | 215 | 1 in 561·32. |
| Cholera, the . . | 4484 | 1 in 26·91. |
| Morbus . . | 699 | 1 in 172·82. |
| Infantum . . | 4870 | 1 in 24·77. |
| Diarrhœa . . | 1606 | 1 in 75·14. |
| Dysentery . . . | 3368 | 1 in 35·83. |
| Marasmus* . . | 4384 | 1 in 27·52. |
| Teething . . . | 2108 | 1 in 57·25. |
| Worms . . . | 997 | 1 in 121·04. |
| Rupture . . . | 121 | 1 in 997·38. |
| Liver, Inflammation and Disease | 1098 | 1 in 109·91. |
| Scirrhus of . . | 115 | 1 in 1049·42. |
| Jaundice . . | 323 | 1 in 373·64. |
| *Genital System.* | | |
| Bladder and Kidneys, Inflammation | 115 | 1 in 1049·42. |
| Gravel or Stone . . | 102 | 1 in 1183·17. |
| Diabetes . . | 28 | 1 in 4310·14. |
| Mismenstruation . . | 37 | 1 in 3261·75. |
| Childbed and Puerperal Fever | 1038 | 1 in 115·3. |
| *Inflammations.* | | |
| Quinsy . . . | 261 | 1 in 462·39. |
| Sore-throat . . | 486 | 1 in 247·49. |
| Rheumatism . . | 195 | 1 in 618·89. |
| Gout . . . | 66 | 1 in 1828·54. |
| White Swelling . . | 59 | 1 in 2045·49. |
| MorbusCoxarius (disease of Hip) | 2 | 1 in 603·42. |
| Abscess . . . | 301 | 1 in 400·91. |
| Ditto, Lumbar . . | 42 | 1 in 2873·42. |
| Tumour . . . | 40 | 1 in 3017·4. |
| Ulcers . . . | 185 | 1 in 652·34. |
| Carbuncle . . . | 26 | 1 in 4641·69. |
| Caries . . . | 41 | 1 in 2943·51. |
| Mortification . . | 447 | 1 in 269·98. |
| Cancer . . . | 358 | 1 in 33·1. |
| Scurvy . . . | 21 | 1 in 5746·9. |
| Rickets . . . | 26 | 1 in 4641·69. |
| Scrofula . . . | 329 | 1 in 366·78. |
| Lues Venerea . . | 372 | 1 in 324·47. |
| Fracture . . . | 115 | 1 in 1049·42. |
| Old Age . . . | 3532 | 1 in 34·19. |
| Drinking Cold Water . | 274 | 1 in 440·45. |
| Intemperance and Delir. Tremens | 1728 | 1 in 69·84. |

* Under deaths from Marasmus are included deaths reported by decay, debility, tabes mesenterica, and atrophy.

| Diseases. | Total Deaths. | Comparative Mortality from each Disease. |
|---|---|---|
| Suicide . . . | 617 | |
| Unknown Disease . | 2472 | 1 in 48·82. |
| *Casualties.* | | |
| Including reports as Burned, Drowned, Frozen, and Killed | 4200 | |
| Malformation* . . | 51 | 1 in 2366·35. |
| Deaths in each year . . | 2297 | |
| Still-born and Premature . | 6925 | |

The whole number of interments for these thirty-two years amounts to 132,426; of which are classed as men, 37,585; as boys, 36,129; women, 28,676; girls, 30,036:—or, males, 73,714; females, 58,712. This amount includes 6,925 still-born, which, in estimating the mortality under five years of age, have been excluded, making an excess of mortality of males of 15,002, or 11·32 per cent. The mortality at certain ages is divided into ten periods, as follows:

| | per Cent. | Total Deaths. |
|---|---|---|
| Under 5 years............ | 39·46 | 49531 |
| Between 5 and 10 years... | 4· | 5023 |
| 10 and 20 ......... | 4·7 | 5899 |
| 20 and 30 ......... | 13·249 | 16628 |
| 30 and 40 ..... ... | 13·462 | 17095 |
| 40 and 50 ...... . | 9·969 | 12512 |
| 50 and 60 ......... | 6·23 | 7785 |
| 60 and 70 ......... | 4·29 | 5385 |
| 70 and 80 ......... | 2·82 | 3541 |
| 80 and upwards.. | 1·674 | 2102 |
| Total ........ | ...... | 125,501 |

The population of this city has risen, during this time, from 75,770 to 270,089; viz.

| | | |
|---|---|---|
| In 1805, | it was | 75,770 |
| In 1810 | .... | 96,373 |
| In 1815 | .... | 100,619 |
| In 1820 | .... | 123,706 |
| In 1825 | .... | 166,086 |
| In 1830 | .... | 197,112 |
| In 1835 | .... | 270,089. |

The rate of mortality, according to the population (still-born excluded), being

In 1805, as 1 to 32·98
In 1810, as 1 to 46·49
In 1815, as 1 to 41·83
In 1820, as 1 to 37·19
In 1825, as 1 to 34·78
In 1830, as 1 to 37·92
In 1835, as 1 to 40·87.

*Remarks on the preceding Table.* By C. A. Lee, M.D.

From this table we are able to learn, with considerable precision, what diseases have increased and what diminished in mortality.

* Malformation includes returns as such, and spina bifida.

In the first place, diseases of the brain seem to have increased nearly fourfold in proportion to the population. Apoplexy nearly one third in the same ratio. Deaths by convulsions, chiefly under one year, have increased nearly threefold; and those from croup, hooping-cough, and inflammation of the lungs, have nearly doubled; while deaths from consumption have increased a little over one third in proportion to population.

Fevers, excluding scarlet and puerperal, have increased only nine per cent.; while scarlet fever has risen from 4 in 1804, to 579 in 1836. The mortality from small-pox is rather less in proportion to the population than in 1801; but measles, which at that time was a rare disease, has now become frequent and fatal, having carried off nearly 500 children in 1835. While inflammation of the stomach and bowels has somewhat increased, cholera infantum is less fatal, by one third, than it was thirty years ago. The same is also true in relation to dysentery and marasmus. As there can be no doubt that, owing to the great extension of the city and the consequent deterioration of air and water, these diseases are more prevalent than they were at that period, it may be reasonably inferred that their diminished mortality is owing to their being treated with greater skill and success than formerly.

Diseases of the genital system have not kept pace with the increase of population, although we now number more than four times the inhabitants we did in 1805; yet the deaths from stone in the bladder are not more numerous than they were at that time, indicating, as we think, more successful methods of cure; for none can deny that the same causes still remain in operation.

Diseases of the heart appear to have increased very much within the last few years. From 1804 to 1834, but nine cases of death from diseases of the heart were reported; since which period they have averaged over twenty annually. Whether this is owing to those causes of mental excitement which have agitated in a remarkable degree all classes of society for the last few years, it may not be possible to determine with certainty; yet no one can doubt that they have exerted considerable influence.

Puerperal fever has kept an even pace with the population, and deaths in childbed have diminished, owing, doubtless, to a better acquaintance with the obstetric art. The whole number of deaths from puerperal fever and in childbed, for thirty-two years, is 1,038; this bearing to the whole number of deaths the ratio of 1 to 115. When we consider the immense number of births which have occurred in this city during the same period, ranging from 2 to 300,000, we shall find that the chances of a fatal result in parturition are small indeed. This result, however, is strikingly at variance with the statement in the Transactions of the Statistical Society of London, viz. that, of 448,356 females who died in the Prussian States in the course of fifteen years, between the ages of fourteen and forty-five, 70,215, or nearly one sixth, died either immediately in the act of delivery or in childbed; and, of the infants born, 1 in 108 cost the mother her life.

The diseases included under the general head of inflammations do not appear to have increased. The deaths from rheumatism have been but 195 during the whole period, and from gout 66. Cancer, scrofula, and syphilis have been of nearly equal fatality, averaging 350 each. The mortality from the venereal disease has, indeed, diminished more than 50 per cent. Whether this indicates a better state of the public morals or an improved *methodus medendi*, we leave for others to judge. The deaths from old age amount to 3,532; being in the ratio of 1 to 34 from deaths from all diseases. While the mortality from drinking cold water has diminished fourfold, that from drinking ardent spirits has increased in the same proportion. The deaths from suicide, in 1805, were 26 in number; in 1836 but 33, instead of 100, as they should have been, to have kept pace with the population. But, perhaps, the most important fact contained in this table is the vast increase in the number of the stillborn and the premature, rising from 47 in 1805 to 506 in 1836, being nearly fivefold in proportion to population. Various causes have been assigned for this increase, none perhaps more plausible than those given by the late talented physician, Dr. Avery, and published in a late Number of this Journal. But, in addition to these, which, it will be recollected, operate chiefly in lowering the standard of female health, thus causing the premature expulsion of the fœtus, there are others, of no slight efficiency; such as the general use of ergot, particularly in difficult and protracted cases of labour. We have witnessed many cases where the death of the fœtus has been caused by the use of this article, and where life might, in all probability, have been saved by an early and judicious use of the forceps. A curious fact connected with this subject is that the number of male stillborn is nearly double that of the female in 1837; the former being 349, and the latter 193. *Amer. Jour. of Med. Sc. May*, 1838.

---

